# Posterior quadratus lumborum versus transversus abdominis plane block for inguinal hernia repair: a prospective randomized controlled study^☆^

**DOI:** 10.1016/j.bjane.2020.11.004

**Published:** 2021-02-10

**Authors:** Onur Okur, Duygu Karaduman, Zeki Tuncel Tekgul, Noyan Koroglu, Mehmet Yildirim

**Affiliations:** a Istanbul Prof. Dr. Cemil Tascioglu City Hospital, Sisli, Istanbul; b Siirt Kurtalan State Hospital, Siirt, Turkey; c Izmir Bozyaka Training and Research Hospital, Izmir, Turkey; d Izmir Katip Celebi University Medical School, Ataturk Training and Research Hospital, Izmir, Turkey

**Keywords:** Postoperative pain, Inguinal hernia, Quadratus lumborum plane block, Transversus abdominis plane block, Spinal anesthesia

## Abstract

**Background and objectives:**

We aimed to compare the analgesic effects of both posterior (type 2) Quadratus Lumborum Block (QLB) and Transversus Abdominis Plane Block (TAPB) compared to spinal anesthesia alone for postoperative pain management in inguinal hernia repair.

**Methods:**

This study enrolled 63 patients scheduled for open inguinal hernia repair. The eligibility criteria were undergoing elective unilateral inguinal hernia repair surgery, having an American Society of Anesthesiologists (ASA) physical status I, II, or III, and not suffering from any chronic pain condition. Group S patients received spinal anesthetics and no additional analgesic treatments. Group T patients received TAPB, and Group Q patients received QLB as analgesic technique in addition to spinal anesthetics.

**Results:**

The pain scores at 6 hours (VAS 6) and 24 hours (VAS 24) were significantly different between groups (*p* < 0.01). Additionally, the sensory and motor block levels were significantly different between groups (*p* < 0.05). Multiple comparison tests showed that patients in Group Q had significantly higher sensory and motor block levels (*p* < 0.01 compared with Group S; *p* < 0.05 compared with Group T). Opioid consumption was significantly different between Groups Q and S (*p* < 0.01) after surgery.

**Conclusions:**

Our findings show that both blocks are similarly effective for the management of postoperative pain compared to spinal anesthesia alone for inguinal hernia repair. We found that QLB resulted in a significant cranial spread compared to TAPB. Opioid consumption in QLB was significantly lower than that in controls but similar to that in TAPB.

## Introduction

The introduction of truncal blocks in clinical practice has changed the management of postoperative pain in abdominal surgery. Regional anesthetic techniques have become an integral part of the mainstay treatment for postoperative pain. In addition to conventional methods of pain control, the use of analgesic plane blocks is encouraged by many associations, including the European Hernia Society.^1^ One of the most pronounced, investigated, and administered techniques for analgesia in abdominal surgeries is the Transversus Abdominis Plane Block (TAPB). Described by Rafi^2^ not more than two decades ago, the TAPB has been shown to be a safe, simple and effective way of alleviating postoperative pain with the addition of ultrasound guidance.^3^ This technique, targeting the transversus abdominis fascia, is effectively performed for analgesia in almost every type of abdominal surgery, such as caesarian sections, laparoscopic procedures, appendectomies, and inguinal hernia repairs.^4^, ^5^, ^6^, ^7^

Another recently popularized analgesic technique is the type 2 Quadratus Lumborum Block (QLB). The QLB, which was first described by Blanco,^8^ is a posterior abdominal wall block targeting the middle thoracolumbar fascia. Previous studies suggest that the quadratus lumborum block is also an effective method of postoperative pain management for abdominal surgeries, such as gynecological procedures, cesarian sections, appendectomies, and colorectal procedures.^9^, ^10^, ^11^ Due to the character of the fascia, dermatomal spread is suggested to be more cranial with the QLB technique than with other abdominal wall blocks.^12^ The underlying mechanism may be related to either lumbar plexus or paravertebral anesthetic distribution.^13^

Studies comparing the effect of TAPB and QLB techniques are scarce and either involve pediatric patients or patients undergoing caesarian section.^14^, ^15^ TAPB has been proven to be effective for acute and chronic pain management in inguinal hernia repair.^7^, ^16^ We hypothesized that QLB in addition to spinal anesthesia would produce superior postoperative analgesia compared to both TAPB in addition to spinal anesthesia and spinal anesthesia alone for inguinal hernia repair surgery. A secondary hypothesis was that QLB would produce a more cranial dermatomal spread of the block compared to TAPB. In this scope, we primarily aimed to compare the analgesic effects of QLB and TAPB to spinal anesthesia alone for postoperative pain management in inguinal hernia repair. Our secondary objectives were to compare the dermatomal spreads, complication rates and amounts of opioid consumption for each block.

## Methods

**Figure 1:**
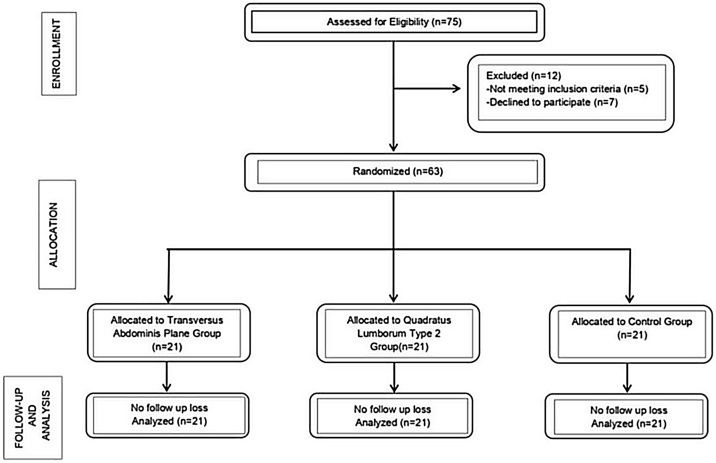
Flow Chart diagram.

This prospective, randomized, controlled, interventional, parallel design study was conducted in accordance with the Helsinki Declaration following approval by the Hospital's Institutional Review Board (IRB #10/11/16-01). The study adheres to applicable CONSORT guidelines. The trial was registered prior to patient enrollment at clinicaltrials.gov (NCT03126084. Principal investigator: O.O. Date of registration: 04/20/2017). Written informed consent was obtained from all subjects participating in the trial. All patients were informed thoroughly about all anesthetic and analgesic techniques in our study, and informed consent was obtained from each patient in addition to study consent. We enrolled 63 patients scheduled for open inguinal hernia repair and allocated them into three groups at a 1:1:1 ratio. Patients were recruited between May and November 2017 and followed until their discharge (Fig. 1). Eligibility criteria were having an elective operation for unilateral inguinal hernia repair, having an American Society of Anesthesiologists (ASA) physical status score of 1, 2 or 3, and not suffering from any chronic pain conditions. The exclusion criteria were patient refusal, allergies to local anesthetic drugs, morbid obesity, hypotension, receiving antithrombolytic treatments, having neurologic sequelae preoperatively, infection in the intended intervention site, or cooperation difficulties (e.g., severe cognitive function disorders).

Patients were enrolled and randomized to one of three groups in ambulatory preoperative evaluation clinics. Random allocation of patients in each group was ensured by the online application “research randomizer” (www.randomizer.org). Patients were allocated to groups according to their enrollment number at the preoperative evaluation clinic. A closed envelope, prepared previously, with the application number inscribed (e.g., #27), was attached to the patient file. The planned intervention was written on cards in the envelope according to the application number (cards read as either TAPB, QLB or N/A). The envelope was opened by the anesthesiologist at the operating room. The anesthesiologist at the operating room (other than those who authored this study) performed the blocks for the study group patients following a subarachnoid block and only performed ultrasound scans of the flank area in the control group patients. Patients in the block groups were blinded to the performed procedure with sterile drapes placed in a way that restricted the patients’ view of the block area. No needle punctures or injections were made in the control group patients. An anesthetic resident who had no information on the performed block collected the patients’ data. One of the authors, who did not participate in the placement of the blocks, performed the data analysis. All interventions were performed in general surgery operating rooms, and patient data were collected in operating rooms, Postanesthesia Care Unit (PACU), and general surgery clinics. Patients were hydrated with 500 mL of normal saline 30–40 minutes prior to surgery. Upon arrival at the operating room, standard ASA monitors were applied to all patients. Lumbar spinal anesthesia via L3–L4 or L4–L5 intervertebral levels with 12.5 mg of hyperbaric bupivacaine 0.5% through a 27G Quincke needle was standard for all groups. The first group (Group S) of patients received no additional analgesic treatments. The second group (Group T) of patients received a transversus abdominis plane block with 2 mg.kg^-1^ of 0.25% plain bupivacaine under ultrasound guidance as an additional analgesic technique. Finally, the third group (Group Q) of patients received a type 2 quadratus lumborum block with 2 mg.kg^-1^ of 0.25% plain bupivacaine under ultrasound guidance as an additional analgesic technique. The sensorial block level was determined as the dermatome where the perception of cold sensation returned upon contact with a freeze pack cooled to 4 °C. Motor block level was assessed according to the Bromage scale (Bromage 0, full flexion of the knee and feet, Bromage 1, just able to move the knees, Bromage 2, just able to move the feet, and Bromage 3, no movement). Sensory spread of the blocks was recorded and compared. Sensory spread was determined by the affected dermatomes with reference to the T10 dermatome (umbilicus). When the sensory block levels were cranial to the T10 dermatome, the affected dermatomes were defined as positive numbers, and when the sensory block levels were caudal to the T10 dermatome, the affected dermatomes were defined as negative numbers. Surgery was initiated when the sensorial block was higher than T10. Patients’ self-reported pain scores were measured by the visual analog scale and recorded immediately after transfer to the Postanesthesia Care Unit (PACU), (VAS 0), 2^nd^ (VAS 2), 4^th^ (VAS 4), 6^th^ (VAS 6), and 24^th^ (VAS 24) postoperative hours. Pain scores were not standardized to rest or movement. Patients whose VAS scores were higher than 5 received 75 mg tramadol as rescue analgesia in addition to 500 mg p.o. paracetamol, and patients were routinely treated with 500 mg paracetamol p.o. every 6 hours. The first rescue administration times and total opioid consumption rates were recorded. PACU or surgical ward nurses recorded all drug administrations, administration times and pain scores (when reported by the patient outside the observation times) on the patient charts. An anesthetic resident recorded measurements within the observation times.

The primary outcome of our study was the difference in VAS score between groups at the 24^th^hour (VAS 24). The secondary outcomes included VAS 0, VAS 2, VAS 4, and VAS 6; the dermatomal spread of local anesthetics; motor block levels; first rescue medication times; and opioid consumption rates.

Statistical analyses were performed with the computerized program Statistical Package for Social Sciences (SPSS version 21, IBM Corporation, Chicago, USA). Continuous variables were reported as the mean and standard deviation, while discrete variables were reported as either number and percent or median and minimum maximum. Normal distribution was tested prior to all analyses with the Shapiro-Wilk’s test. Normally distributed quantitative data were compared using one-way ANOVA. Tukey’s HD test was used for post-hoc analysis whenever necessary. Nonnormally distributed quantitative data were compared with the Kruskal-Wallis H test. The Mann-Whitney *U* test was used for post-hoc analysis whenever necessary. Qualitative data were compared using contingency table analyses. A multiple regression approach to contingency analysis (with Bonferroni correction) was used for post-hoc analysis. The significance of the change between repeated measurements was analyzed with the Friedman test. The significance was interpreted when *p*-values were lower than 0.05 unless stated otherwise. Sample size calculation was performed with the computer-based power analysis program G-power (v. 3). The minimum sample size of 21 subjects per group was determined by assuming an α error of 0.05 and a power of 80% for an effect size of 0.4109609 depending on a pilot study on 15 subjects. Sample size calculation was based on a pain score comparison at 24 hours.

## Results

Sixty-three patients were randomly assigned into three groups, and all of the patients received the intended treatments and were analyzed for the primary outcome. Demographics are presented in Table 1. Analysis showed that the age, sex, surgery duration and ASA physical status score data of the patients did not follow a normal distribution. On the other hand, the body weight and height data followed a normal distribution. Comparison of the demographics and ASA status scores between groups showed no statistically significant difference (*p* > 0.05).

**Table 1:** Demographics of the patients.

	**Group S**	**Group T**	**Group Q**	***p*-values**
**Gender (M/F) (n)**	19/9	19/9	21/0	0.344^a^
**Age (years) (mean ± SD)**	60.9 ± 13.8	50.7 ± 17.8	58.1 ± 16.6	0.114^b^
**Weight (kg) (mean ± SD)**	76.6 ± 7.6	73.4 ± 13.4	76 ± 11.8	0.563^c^
**Duration of surgery (min) (mean ± SD)**	89.8 ± 30.5	89.8 ± 29.9	92.1 ± 20.4	0.571^b^
**ASA (I/II/III) (n)**	2/19/0	6/15/0	2/17/2	0.104^a^

ASA, American Society of Anesthesiologists; F, Female; M, Male; n, number; SD, Standard Deviation; *p* < 0.05.

a Contingency table analysis.

b Kruskal-Wallis H test.

c ANOVA.

We evaluated the patients’ postoperative pain scores at 0, 2, 4, 6, and 24 hours and found that the data were not normally distributed. There were no significant differences in the VAS 0, VAS 2, and VAS 4 pain scores between groups (*p* = 0.368, *p* = 0.064, *p* = 0.100, respectively). However, we found a statistically significant difference in VAS 6 and VAS 24 between groups (*p* < 0.001, *p* < 0.001). Multiple comparisons showed that Group T and Group Q patients had significantly lower pain at 6 and 24 hours compared to controls (*p* = 0.010, *p* < 0.001 for Group T and *p* < 0.001, *p* < 0.001 for Group Q) (Table 2). We failed to show any difference between Group T and Group Q patients in terms of the VAS 6 and VAS 24 pain scores (*p* = 0.148, *p* = 0.673, respectively).

**Table 2:** Comparison of postoperative VAS scores between groups.

	**Group S (mean ± SD)**	**Group T (mean ± SD)**	**Group Q (mean ± SD)**	***p*-values**
**Postoperative 0**	0.10 ± 0.44	0 ± 0	0 ± 0	0.368^a^
2^nd^ hour	0.67 ± 0.97	0.38 ± 0.80	0.95 ± 0.44	0.064^a^
4^th^ hour	0.73 ± 1.23	0.71 ± 1.35	0.33 ± 0.80	0.100^a^
6^th^ hour	1.14 ± 1.39	0.90 ± 1.14	0.43 ± 0.81	< 0.001^a^
Group S vs. Group T				0.010^b^
Group S vs. Group Q				< 0.001^b^
Group Q vs. Group T				0.150^b^
24^th^ hour	1.90 ± 0.77	0.95 ± 0.74	0.85 ± 0.73	< 0.001^a^
Group S vs. Group T				< 0.001^b^
Group S vs. Group Q				< 0.001^b^
Group Q vs. Group T				0.67^b^

VAS, Visual Analogue Pain Scale in cm; SD, standard deviation; *p* < 0.05.

a Kruskal-Wallis H test.

b Mann-Whitney U test for multiple comparison analysis.

We tested the difference in sensory and motor spread of spinal anesthesia between groups and found a significant difference between groups (*p* = 0.008, *p* = 0.021). Multiple comparisons revealed that Group Q patients had significantly higher sensory and motor spread of the block than Group S patients (*p* = 0.005, *p* = 0.049), and Group T patients (*p* = 0.01, *p* = 0.025). Group Q patients had a mean dermatomal spread of 3.14 ± 1.53 dermatomes, which was higher than both Group T (1.52 ± 2.14) and Group S (1.62 ± 1.40) patients. Motor spread was determined by Bromage scale scores. The number of Bromage 3 patients was 14 in Group Q, 8 in Group T and 7 in Group S. We found that the length of surgery and the length of hospital stay were similar between groups. The number of comorbidities was also similar between groups.

Time to first pain report by patients were recorded. Only one patient from Group S reported pain before VAS 0 measurement. First, pain reporting times were significantly longer in Group T and Q patients than in Group S patients (*p* = 0.014, *p* < 0.001), but there was no statistically significant difference between Group T and Q patients (*p* = 0.128).

Although there was no significant difference in opioid use (e.g., yes/no) between groups (*p* = 0.080), opioid consumption amount was significantly different (*p* = 0.029). Multiple comparisons further demonstrated that the difference in opioid consumption resulted from comparison of groups Q and S (*p* = 0.007). No other statistically significant differences were observed in comparisons between the other groups. (*p* = 0.299 for Group Q and T and, *p* = 0.148 for Group T and Group S).

Analysis of complications showed that significantly more Group T patients suffered from Post Dural Puncture Headache (PDPH), (*p* = 0.0019; where the limit of significance was corrected to *p* < 0.0083), while bradycardia (*p* = 0.0009; limit of significance *p* < 0.0083) and hypotension (*p* = 0.0069; limit of significance *p* < 0.0083) rates were significantly higher in Group Q patients. There were no complications related to block administration (e.g., hematoma, organ perforation/pneumothorax, local anesthetic systemic toxicity, allergic reaction).

## Discussion

This randomized controlled study compared the analgesic effectiveness of posterior QLB and TAPB in addition to spinal anesthesia and spinal anesthesia alone for pain management in inguinal hernia repair. Our findings of the current study indicate that both blocks are similarly effective and superior to spinal anesthesia alone in ameliorating postoperative pain for inguinal hernia repair. Additionally, we found that QLB resulted in a significantly more cranial spread compared to TAPB, possibly due to potentiation of spinal anesthesia.

Although both QLB and TAPB are commonly used for analgesia in abdominal surgery, previous evidence shows that QLB provides better analgesia than TAPB. Blanco et al. compared patients undergoing caesarian section and found that QLB was superior for ameliorating both pain scores and opioid requirements.^15^ Another study by Oksuz et al. comparing QLB and TAPB in pediatric patients showed better pain scores in the QLB group.^14^ Our study failed to produce similar results. Pain scores were significantly lower compared to the spinal anesthesia group, but there were no significant differences between block groups. Although QLB significantly reduced opioid consumption compared to controls, a similar reduction was not observed between QLB and TAPB.

Elsharkawy et al.^13^ suggested that dermatomal spread with QLB, while still more cranial than TAPB, is stable and predictable. However, our study showed that the combination of QLB with spinal anesthesia increased its dermatomal spread, creating less predictable cranial spread. Our findings showed that significantly more patients suffered from bradycardia and hypotension in the QLB group of patients. We believe this is an indirect result due to potentiation of the subarachnoid block. Evidence supports this suggestion, as higher dermatomal spread was observed in the QLB group of patients regardless of similar height, age, and local anesthetic dosing. This might be due to a better paravertebral spread of local anesthetic, nerve root involvement or synergism of thoracolumbar fascial spread with QLB, as implied by other studies.^9^, ^12^, ^13^, ^17^ This finding indicates that side effects related to the administration of QLB alongside spinal anesthesia in elderly or critically ill patients might be poorly tolerated. Therefore, when dealing with such patients, extra measures should be taken, such as the preparation of inotropes and fluids, dose reduction for spinal anesthesia, avoidance of the combination of QLB and spinal anesthesia, and the use of QLB as the sole anesthetic for inguinal hernia repair surgery.

This study followed a rigorous randomization and blinding protocol in order to limit potential bias. Patients were blinded to the block (TAPB/QLB) and authors were not allowed to perform those interventions. However, the assistant staff who were responsible for anesthetic blocks administration were aware of treatments. Additionally, we used subarachnoid block as a primary anesthetic technique, and this might be a source of imprecision for pain evaluation early after surgery due to residual spinal anesthesia. Moreover, pain score assessments were not standardized to rest or movement, which might be a limitation of the present study. Finally, another limitation may be related to the absence of monitoring long-term pain scores and chronic pain after surgery.

In conclusion, our results indicate that both TAPB and QLB provide adequate postoperative analgesia for adult patients undergoing inguinal hernia repair. Both blocks were found to be superior to spinal anesthesia alone for postoperative pain management. However, there were no significant differences in postoperative pain scores between the QLB and TAPB groups of patients. Both TAPB and QLB are similarly effective ways to manage postoperative pain in inguinal hernia repair; therefore, factors other than pain control should play a role in the choice between these two blocks. As a secondary conclusion, our findings show that the administration of QLB alongside spinal anesthesia produces more frequent side effects, such as bradycardia and hypotension, due to the potentiation of spinal block and might be poorly tolerated, especially in elderly and critically ill patients. Greater vigilance might be crucial in such patients, and the choice of QLB as the sole anesthetic for inguinal hernia repair might prove more beneficial.

## Conflicts of interest

The authors declare no conflicts of interest.

## References

[1] Simons M., Aufenacker T., Bay-Nielsen M. (2009). European hernia guidelines on the treatment of inguinal hernia in adult patients. Hernia.

[2] Rafi A. (2001). Abdominal field block: A new approach via the lumbar triangle. Anaesthesia.

[3] McDonnell J.G., O’Donnell B., Curley G. (2007). The analgesic efficacy of transversus abdominis plane block after abdominal surgery: A prospective randomized controlled trial. Anesth Analg.

[4] McDonnell J.G., Curley G., Carney J. (2008). The analgesic efficacy of transversus abdominis plane block after cesarean delivery: a randomized controlled trial. Anesth Analg.

[5] El-Dawlatly A.A., Turkistani A., Kettner S.C. (2009). Ultrasound-guided transversus abdominis plane block: description of a new technique and comparison with conventional systemic analgesia during laparoscopic cholecystectomy. Br J Anaesth.

[6] De-Oliveira G.D., Castro-Alves L.J., Nader A. (2014). Transversus abdominis plane block to ameliorate postoperative pain outcomes after laparoscopic surgery: A meta-analysis of randomized controlled trials. Anesth Analg.

[7] Venkatraman R., Abhinaya R.J., Sakthivel A. (2016). Efficacy of ultrasound-guided transversus abdominis plane block for postoperative analgesia in patients undergoing inguinal hernia repair. Local Reg Anesth.

[8] Blanco R. (2007). Tap block under ultrasound guidance: the description of a “no pops” technique: 271. Reg Anesth Pain Med.

[9] Blanco R., Ansari T., Girgis E. (2015). Quadratus lumborum block for postoperative pain after cesarean section. Eur J Anaesthesiol.

[10] Ishio J., Komasawa N., Kido H. (2017). Evaluation of ultrasound-guided posterior quadratus lumborum block for postoperative analgesia after laparoscopic gynecologic surgery. J Clin Anesth.

[11] Akerman M., Pejčić N., Veličković I. (2018). A review of the quadratus lumborum block and ERAS. Front Med.

[12] Carney J., Finnerty O., Rauf J. (2011). Studies on the spread of local anaesthetic solution in transversus abdominis plane blocks. Anaesthesia.

[13] Elsharkawy H., El-Boghdadly K., Kolli S. (2017). Injectate spread following anterior sub-costal and anterior approaches to the quadratus lumborum. Eur J Anaesthesiol.

[14] Oksuz G., Bilal B., Gurkan Y. (2017). Quadratus lumborum block versus transversus abdominis plane block in children undergoing low abdominal surgery: A randomized controlled trial. Reg Anesth Pain Med.

[15] Blanco R., Ansari T., Riad W. (2016). Quadratus lumborum block versus transversus abdominis plane block for postoperative pain after cesarean delivery: A randomized controlled trial. Reg Anesth Pain Med.

[16] Okur O., Tekgul Z.T., Erkan N. (2017). Comparison of efficacy of transversus abdominis plane block and iliohypogastric/ilioinguinal nerve block for postoperative pain management in patients undergoing inguinal herniorrhaphy with spinal anesthesia: A prospective randomized controlled open-label study. J Anesth.

[17] Ueshima H., Otake H., Lin J.A. (2017). Ultrasound-guided quadratus lumborum block: An updated review of anatomy and techniques. Biomed Res Int.

